# Johnson Cook Material and Failure Model Parameters Estimation of AISI-1045 Medium Carbon Steel for Metal Forming Applications

**DOI:** 10.3390/ma12040609

**Published:** 2019-02-18

**Authors:** Mohanraj Murugesan, Dong Won Jung

**Affiliations:** Department of Mechanical Engineering, Jeju National University, Jeju-Do 63243, Korea; mohanaero45@gmail.com

**Keywords:** AISI-1045 medium carbon steel, flow stress, surrogate modeling, Johnson–Cook material and damage model, metal forming simulations

## Abstract

Consistent and reasonable characterization of the material behavior under the coupled effects of strain, strain rate and temperature on the material flow stress is remarkably crucial in order to design as well as optimize the process parameters in the metal forming industrial practice. The objective of this work was to formulate an appropriate flow stress model to characterize the flow behavior of AISI-1045 medium carbon steel over a practical range of deformation temperatures (650–950 ${}^{\circ }$C) and strain rates (0.05–1.0 s${}^{-1}$). Subsequently, the Johnson-Cook flow stress model was adopted for modeling and predicting the material flow behavior at elevated temperatures. Furthermore, surrogate models were developed based on the constitutive relations, and the model constants were estimated using the experimental results. As a result, the constitutive flow stress model was formed and the constructed model was examined systematically against experimental data by both numerical and graphical validations. In addition, to predict the material damage behavior, the failure model proposed by Johnson and Cook was used, and to determine the model parameters, seven different specimens, including flat, smooth round bars and pre-notched specimens, were tested at room temperature under quasi strain rate conditions. From the results, it can be seen that the developed model over predicts the material behavior at a low temperature for all strain rates. However, overall, the developed model can produce a fairly accurate and precise estimation of flow behavior with good correlation to the experimental data under high temperature conditions. Furthermore, the damage model parameters estimated in this research can be used to model the metal forming simulations, and valuable prediction results for the work material can be achieved.

## 1. Introduction

Understanding the damage caused by plastic deformation in the metal forming process is essential to make safe the operation of structures in the working field as well as to reduce the cost and time consumption of the experiments. In industrial practice, the Johnson–Cook (JC) material and damage model is extensively incorporated into most of the available finite element (FE) tools to model metal forming simulations because of its ability to predict the model parameters with less effort. It is undeniable that the well-made and reliable proposed flow stress model is more supportive over a wide range of strain rates and elevated temperatures for product design in terms of predicting the material ductility behavior efficiently. Even though the flow stress models are broken down into different categories, such as physically-based, empirical, and semi-empirical, the aim of these models to achieve accurate prediction of the material behavior for a specific material remains the same [1]. So, developing a proper flow stress model for the design process is essential to predict material deformation behavior at high strain rates and deformation temperatures, and, as a result, reasonable research has been performed considering various materials.

Aviral Shrot et al. [2] proposed a method using the Levenberg-Marquardt search algorithm for the inverse identification of JC material parameters. A set of JC parameters was used to develop an idealized FE model for the machining process. Then, the inverse identification method was used to estimate the JC by looking at the chip morphology and the cutting force during the process. They concluded that it is possible to re-identify the model parameters by inverse methods; however the estimated parameters from the simulation results were almost identical to the original set. Luca Gambirasio et al. [3] adopted various procedures for calibrating the JC model parameters under the high strain rate phenomenon by expressing the deviatoric behavior of elasto-plastic materials. In addition, the Taylor impact test experimental data was also included for the evaluation of the JC strength model parameters. The same authors, Luca Gambirasio et al. [4], introduced a new strength model, named split JC, for the flow stress prediction of three real materials. They stated that the split JC model provides a much more improved coherence on the plastic material description and the capability to predict the material behavior is remarkably better that the original JC. Ravindranadh Bobbili et al. [5] employed an artificial neural network (ANN) constitutive model and the JC model to predict the material’s behavior at a high strain rate generated from split Hopkinson pressure bar (SHPH) experiments at various temperatures for 7017 aluminium alloy. It was reported that the predictions of the ANN model were observed to be more consistent with the experimental data than the JC model for all strain rates and temperatures.

A. S. Milani et al. [6] used a weighted multi-objective identification strategy to estimate JC material parameters of two materials, Nitronic 33 super alloy and Ti-6Al-4V, which are often used in models of FE tools. The proposed framework was found to be admirable in terms of reducing the number of experiments necessary for the identification of model parameters. G. H. Majzoobi et al. [7] adopted an optimization approach instead of conducting conventional experiments which are time and cost consuming. They concluded that JC material and failure model parameters were estimated, and a good agreement between experimental and FE predictions was observed. A. Banerjee et al. [8] investigated the typical behavior of armour steel material to analyze and predict its response to various dynamic loading conditions. The developed JC constitutive and damage model displayed a reasonable agreement between the Charpy impact test simulation and experimental results. A. E. Buzyurkin et al. [9] investigated the fracture behaviour of titanium alloys based on the available experimental data obtained from various dynamic loading conditions. The model parameters of the JC model were determined and incorporated into the FE simulations to solve a case of an aircraft engine fan problem. The simulation results of the fan case provided better agreement with the experimental data. Xueping Zhang et al. [10] examined the hard turning process to predict the effects of cutting parameters and tool geometry using the JC damage model, and the results showed better agreement with the experiments. Jian Liu et al. [11] evaluated a metal cutting process with six ductile fracture models to identify the appropriate fracture model. They stated that suitable damage prediction was obtained from the Bao-Wierzbicki fracture model, including rate dependency, temperature effect and damage evolution.

From a literature survey, it was identified that constructing a proper FE model typically requires expensive experimental effort, and appropriate modeling of fracture behavior is necessary, and the damage model should consider both damage initiation and damage evolution. Even though many researchers were worked with the original JC model, only very few researchers were reported about the strategy to optimize the JC model constants to improve the model predictability. Furthermore, so far, there has been no attempt to develop a detailed JC material and damage model for AISI-1045 medium carbon steel material. The aim of this research was to identify the most consistent JC constitutive and damage model parameters for AISI-1045 medium carbon steel material, and, in addition, to exploit an empirical model approach in order to fit the constitutive equation using experimental data. Isothermal tensile tests were carried out at elevated temperatures (650–950 ${}^{\circ }$C) and at low strain rates (0.05–1.0 s${}^{-1}$) to determine the model properties. To improve the predictability of the JC material model, an optimization procedure based on the nonlinear programming solver, find minimum of constrained nonlinear multivariable function (fmincon), was employed, considering the strain rate hardening and the thermal softening parameters. Also, an extensive series of experiments, including un-notched and pre-notched flat and round bar specimens, were conducted at quasi-static strain rates (0.0001–0.05 s${}^{-1}$) to estimate the damage model parameters of the JC model.

## 2. Experimental Procedures

**Figure 1:**
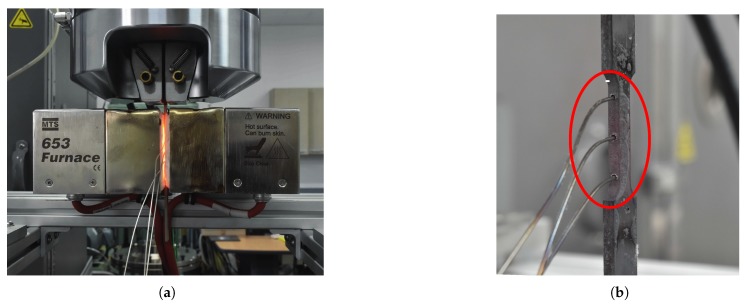
Experimental set-up. (**a**) Test machine; (**b**) Specimen with thermocouples.

**Figure 2:**
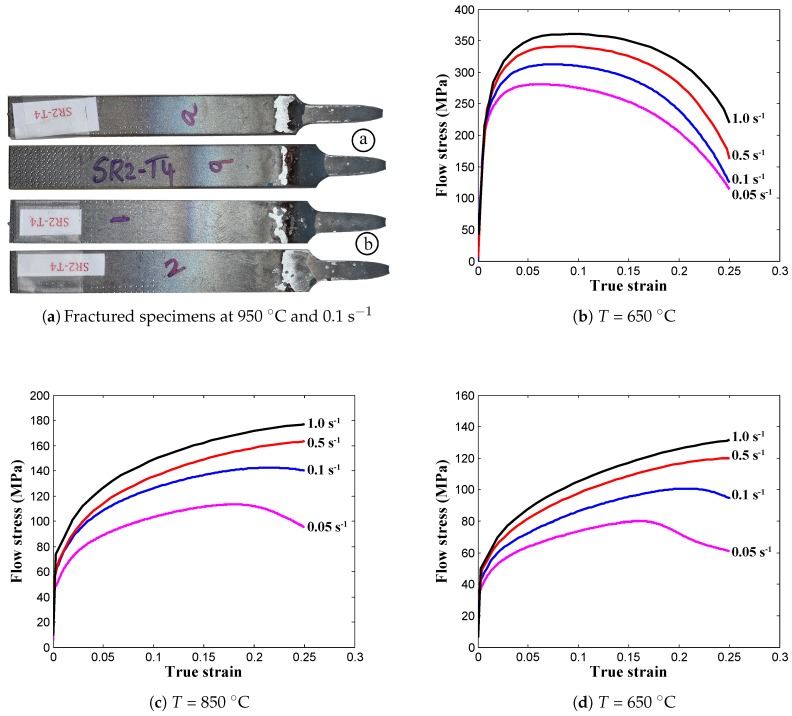
True strain-true stress data obtained from hot tensile tests at various temperatures under different strain rates.

The AISI-1045 medium carbon steel material was investigated in the present research work, and the chemical composition (in wt.%) of the steel is listed in Table 1. The specimens were prepared by the water jet cutting process from the AISI-1045 steel plates and were further used in uniaxial tensile tests to obtain the flow stress-strain data to characterize the hot deformation flow behavior. In detail, the tensile test specimens were prepared with a gauge length of 25 mm and a thickness of 3 mm, according to the ASTM-E8M-subsize standard. Tensile tests were performed at elevated deformation temperatures (650–950 ${}^{\circ }$C) and high strain rates (0.05–1.0 s${}^{-1}$) on a computer-controlled servo-hydraulic testing machine, as shown in Figure 1a, which can heat the specimen to a maximum of 950 ${}^{\circ }$C. From Figure 1a, it can be seen that the clamped tensile specimen was covered with the isolation part to achieve isothermal conditions, and, in addition, a detailed view of the prepared tensile specimen, which was inside the testing machine, is shown in Figure 1b. Before conducting the tests, as displayed in Figure 1b, the calibrations were done using the thermocouples to determine the heating time to obtain an approximately uniform temperature distribution for the specific temperature value, and then, the noted details were used to conduct the experiments. During the experiment, two specimens were tested for each case, and the averaged load-stroke data were converted into the true stress-strain data using the standard equations of the simple tensile tests. The fractured specimens and the obtained flow stress-strain data are displayed in Figure 2. Subsequently, the elastic region was removed from the flow stress-strain curve in order to get the true plastic flow stress-strain data for the purpose of estimation of the constitutive model parameters.

## 3. Johnson-Cook Model

The metallic material relationships between stress and strain can be described by the Johnson-Cook model under the conditions of large deformation, high strain rate and elevated temperatures. Being in a simple form and as it requires less effort to estimate the material constants, it has been widely employed by many researchers to predict the flow behavior of materials. The flow stress model is expressed as follows [12,13,14,15,16,17]:

(1) $$\sigma =\left(A+B\varepsilon^{n}\right)\left(1+C\;\ln{\dot{\varepsilon }}^{*}\right)\left(1-{T^{*}}^{m}\right),$$

where σ is the equivalent stress, and ε is the equivalent plastic strain. The material constants are *A*, *B*, *n*, *C* and *m*. *A* is the yield stress of the material under reference conditions, *B* is the strain hardening constant, *n* is the strain hardening coefficient, *C* is the strengthening coefficient of strain rate, and *m* is the thermal softening coefficient [18].

The three parenthesis components in Equation (1) represent, from left to right, the strain hardening effect, the strain rate strengthening effect and the temperature effect, which influences the flow stress values [19]. In the flow stress model, ${\dot{\varepsilon }}^{*}$ and $T^{*}$ are

$${\dot{\varepsilon }}^{*}=\frac{\dot{\varepsilon }}{{\dot{\varepsilon }}_{\mathrm{ref}}},\;T^{*}=\frac{T-T_{\mathrm{ref}}}{T_{m}-T_{\mathrm{ref}}},$$

where ${\dot{\varepsilon }}^{*}$ is the dimensionless strain rate, $T^{*}$ is the homologous temperature, $T_{m}$ is the melting temperature of the material, and *T* is the deformation temperature. ${\dot{\varepsilon }}_{\mathrm{ref}}$ and $T_{\mathrm{ref}}$ are the reference strain rate and the reference deformation temperature, respectively [19]. In the present study, regarding the experimental conditions, the reference strain rate, ${\dot{\varepsilon }}_{\mathrm{ref}}$, and the reference temperature, $T_{\mathrm{ref}}$, were taken as 1223 K and 1 s${}^{-1}$, respectively. In accordance with the reference conditions, the material constant, *A*, was determined to be 50.103 MPa for further calculations.

### 3.1. Determination of Material Constants B and n

When the deformation temperature is *T* = $T_{ref}$ = 1223 K, and the deformation strain rate is $\dot{\varepsilon }={\dot{\varepsilon }}_{ref}$ = 1 s${}^{-1}$, Equation (1) is modified as follows [19]:

(2) $$\sigma =(A+B\varepsilon^{n}).$$

Here, the influences of strain rate strengthening and thermal softening effects are neglected. By rearranging Equation (2) and taking the natural logarithm on both sides of Equation (2), the modified equation can be obtained as shown below [19]:

(3) ln(σ−A)=nlnε+lnB.

**Figure 3:**
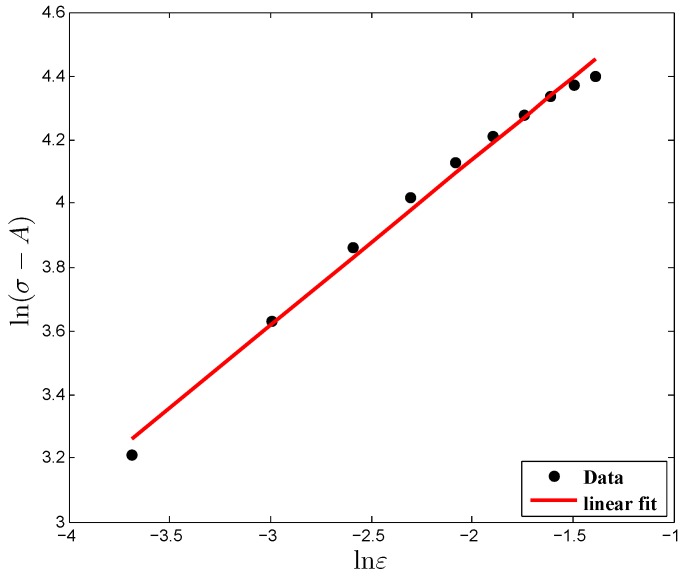
Relationship between ln(σ−A) and lnε under the reference conditions.

By substituting the flow stress and strain values at the reference deformation conditions into Equation (3), the linear relationship plot between ln(σ−A) and lnε was drawn, and then the first-order regression model was used to fit the data points as depicted in Figure 3. From Figure 3, it is noted that more than 96 percent of the data lies very close to the regression line, which shows the better predictability of the data distribution. As a result, the material constants *B* and *n* were estimated to be 176.09 MPa and 0.5176 from the slope and intercept of the fitted curve.

### 3.2. Determination of Material Constant C

When the deformation temperature is *T* = $T_{ref}$ = 1223 K, Equation (1) can be remodeled as shown below [19]:

(4) $$\sigma =\left(A+B\varepsilon^{n}\right)\left(1+C\;\ln{\dot{\varepsilon }}^{*}\right),$$

whereas, the influences of thermal softening effects are ignored. Rearranging Equation (4) will result in the following form:

(5) $$\frac{\sigma }{\left(A+B\varepsilon^{n}\right)}=\left(1+C\;\ln{\dot{\varepsilon }}^{*}\right).$$

**Figure 4:**
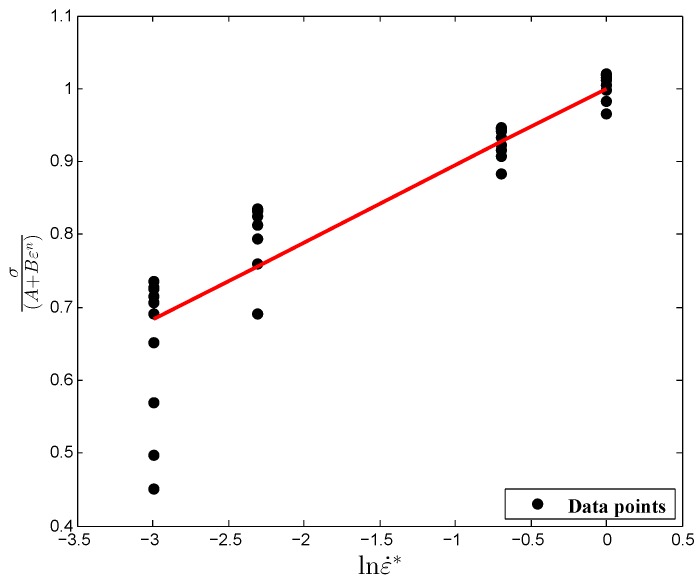
Relationship between $\frac{\sigma }{\left(A+B\varepsilon^{n}\right)}$ and $\ln{\dot{\varepsilon }}^{*}$ under the reference conditions.

Initially, the values of material constants *A*, *B* and *n*, obtained in Section 3.1, were substituted into Equation (5); then, $\frac{\sigma }{\left(A+B\varepsilon^{n}\right)}∼\ln{\dot{\varepsilon }}^{*}$ was drawn as a curve, as shown in Figure 4. Subsequently, linear fitting was carried out using the first-order regression model with an intercept value of 1, considering flow stress values at four strain rates (0.05 s${}^{-1}$, 0.1 s${}^{-1}$, 0.5 s${}^{-1}$ and 1 s${}^{-1}$). Finally, the slope of the fitting curve, the material constant value, *C*, was estimated to be 0.1056. Here, it is important to mention that at first, the material constant, *C*, was estimated based on the traditional method considering all strain values. However, in this research, the optimization procedure was adopted to find out the optimum *C* value in order to reduce the prediction error compared with the experimental data. For this purpose, the material constant, *C*, was estimated at ten discrete strain values, and, as a result, ten different values of *C* were obtained from the fitted linear model. Thereafter, the estimated values were further used in the optimization calculations.

### 3.3. Determination of the Material Constant, m

When the deformation strain rate is $\dot{\varepsilon }={\dot{\varepsilon }}_{ref}$ = 1 s${}^{-1}$, Equation (1) can be simplified as [19]:

(6) $$\sigma =\left(A+B\varepsilon^{n}\right)\left(1-{T^{*}}^{m}\right).$$

Here, the influences of the strain rate strengthening effect are neglected. Equation (6) is rearranged into the following form:

(7) $$1-\frac{\sigma }{\left(A+B\varepsilon^{n}\right)}={T^{*}}^{m}.$$

Taking the natural logarithm on both sides of Equation (7), the following equation can be obtained as:

(8) $$\ln\left[1-\frac{\sigma }{\left(A+B\varepsilon^{n}\right)}\right]=m\;\ln T^{*}.$$

**Figure 5:**
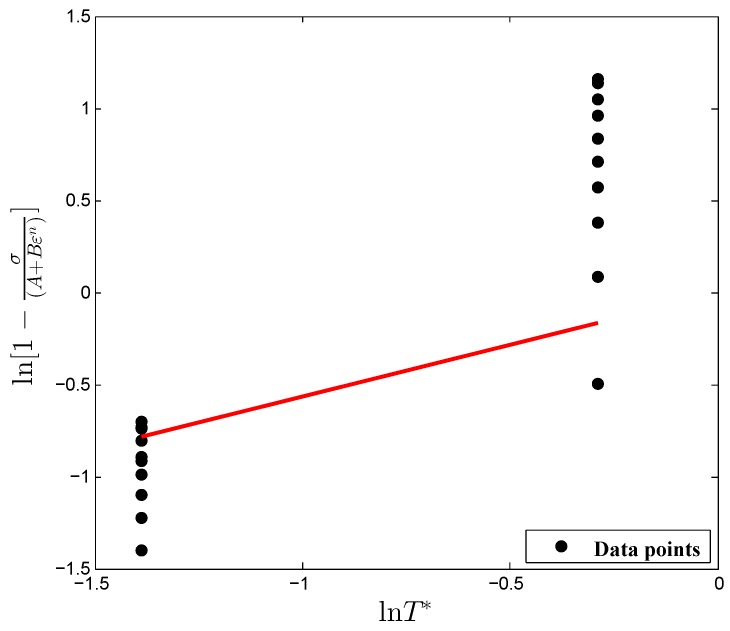
Relationship between $\ln\left[1-\frac{\sigma }{\left(A+B\varepsilon^{n}\right)}\right]$ and $\ln T^{*}$ under the reference conditions.

**Figure 6:**
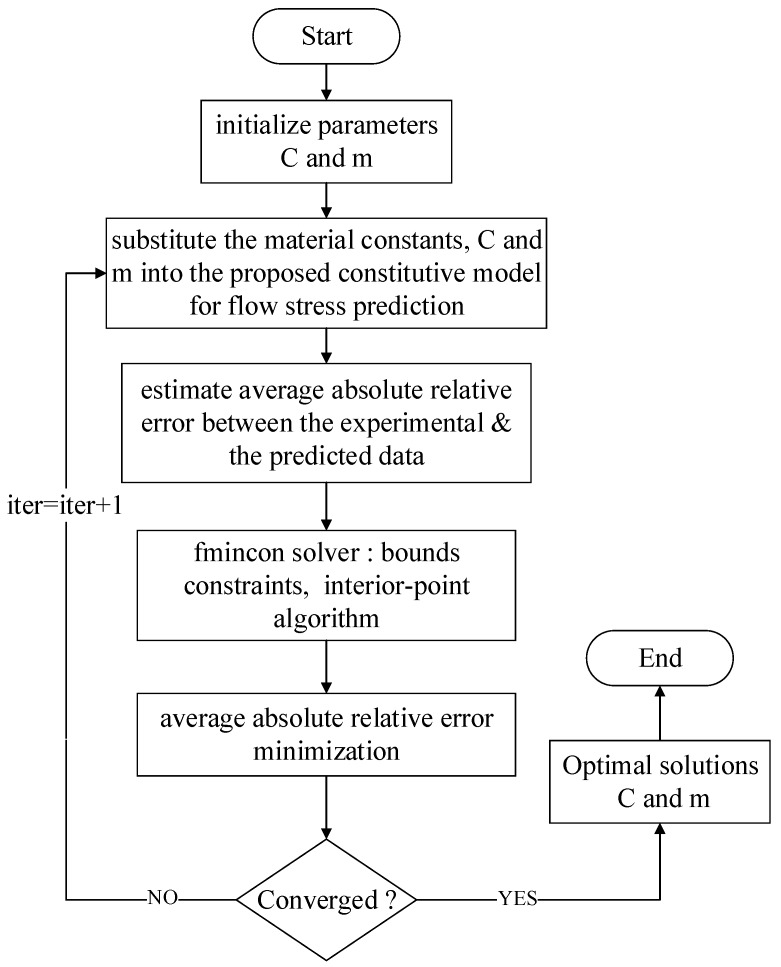
Flow chart of the optimization procedure to find the minimum of an objective function in the presence of bound constraints.

Substituting the values of material constants *A*, *B* and *n* into Equation (8) and fitting the data points using the first-order regression model, as shown in Figure 5, the material constant, *m*, was determined to be 0.5655 from the slope of fitted curve considering the conventional method. As with the material constant, *C*, from the estimation at ten discrete strain points, ten different *m* values were obtained from the flow stress values of two different temperatures (923 K, 1123 K) for optimization purposes. Hereafter, a bounds constrained optimization procedure (Figure 6) was used to find the optimum solution of the material constants *C* and *m*, and the optimization formulation used in the present work is expressed below:

$$\begin{array}{c} \left\{\begin{array}{c} \underset{x}{\mathrm{Minimize}:\;}\\ \;\;\mathrm{AARE}=\frac{1}{n}\sum_{i=1}^{n}\left|\frac{\sigma_{\exp}^{i}-\sigma_{\mathrm{pred}}^{i}}{\sigma_{\exp}}\right|\times 100\%,\\ \mathrm{where},\sigma_{\mathrm{pred}}=\left(A+B\varepsilon^{n}\right)\left(1+x\left(1\right)\ln{\dot{\varepsilon }}^{*}\right)\left(1-{T^{*}}^{x(2)}\right)\\ \mathrm{subjected}\;\mathrm{to}\;\;\left\{\begin{array}{c} \;C_{\min}\leq x\left(1\right)\leq C_{\max}\\ \;m_{\min}\leq x\left(2\right)\leq m_{\max} \end{array}\right. \end{array}\right. \end{array}$$

To solve this optimization problem, the nonlinear programming solver, fmincon, was used with the interior-point (IP) algorithm to minimize the average absolute relative error between the experimental data and the predicted data. The main reason to use the IP algorithm was because the aim was to find the minimum of an objective function in the presence of bound constraints alone. The optimization problem was solved after 29 successful iterations with the function value, the minimized prediction error, 17.62%, and the optimum solutions for the material constants *C* and *m* were 0.095 and 0.6622, respectively. The material constants, which were estimated from the constitutive equations and the optimization procedure of the proposed JC model, are listed in Table 2.

Thus, the material constants were substituted into Equation (1) to form a final flow stress model. Thereafter, the relations among stress, σ, strain, ε, the deformation strain rate, $\dot{\varepsilon }$, and the deformation temperature, *T*, were established according to the JC model, as follows:

(9) $${\hat{\sigma }}_{\mathrm{pred}}=\left(50.103+176.09\varepsilon^{0.518}\right)\left(1+0.095\;\ln\left(\frac{\dot{\varepsilon }}{1.0}\right)\right)\left(1-{\left(\frac{T-1223}{1623-1223}\right)}^{0.662}\right)\;\left(\mathrm{MPa}\right).$$

### 3.4. Johnson-Cook Damage Model

Johnson and Cook proposed that fracture strain typically depends on the stress triaxiality ratio, the strain rate and the temperature. The JC fracture model can be written as follows [8,20]:

(10) $$\varepsilon_{f}=\left[D_{1}+D_{2}\exp\left(D_{3}\left(\frac{\sigma_{m}}{\sigma_{\mathrm{eq}}}\right)\right)\right]\left[1+D_{4}\ln\left({\dot{\varepsilon_{p}}}^{*}\right)\right]\left[1+D_{5}T^{*}\right]$$

where $D_{1}$ to $D_{5}$ are the damage model constants, $\sigma_{m}$ is the mean stress, and $\sigma_{\mathrm{eq}}$ is the equivalent stress [19]. The damage of an element is defined based on a cumulative damage law, and it can be represented in a linear way as shown below [9,19]:

(11) $$D=\sum \left(\frac{\Delta \varepsilon }{\varepsilon_{f}}\right),$$

where Δε is the equivalent plastic strain increment, and $\varepsilon_{f}$ is the equivalent strain to fracture under the present conditions of stress, strain rate and temperature. Due to the fracture occurrence, the material strength reduces during deformation, and the constitutive relation of stress for the damage evolution can be expressed as [8]

(12) $$\sigma_{D}=\left(1-D\right)\sigma_{\mathrm{eq}.}$$

In Equation (13), $\sigma_{D}$ is the damaged stress state, and *D* is the damage parameter, (0≤D<1). Furthermore, the stress triaxiality [21,22,23,24,25] and the equivalent stress can be obtained from undamaged material considering the plastic behavior until the necking formation [19].

**Figure 7:**
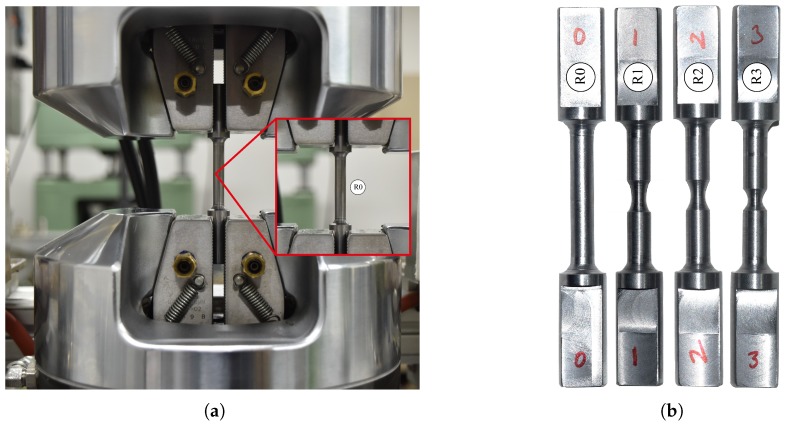
Experimental set-up to perform tension test at room temperature. (**a**) Test machine; (**b**) Un-notched and pre-notched specimens.

**Figure 8:**
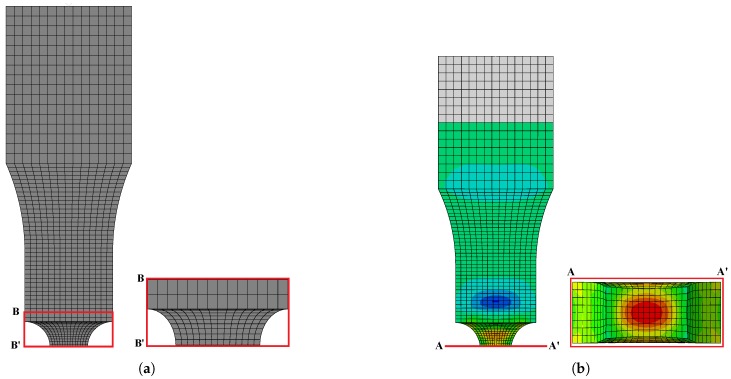
Finite element models. (**a**) Fine mesh in notching region; (**b**) Stress estimation region.

**Figure 9:**
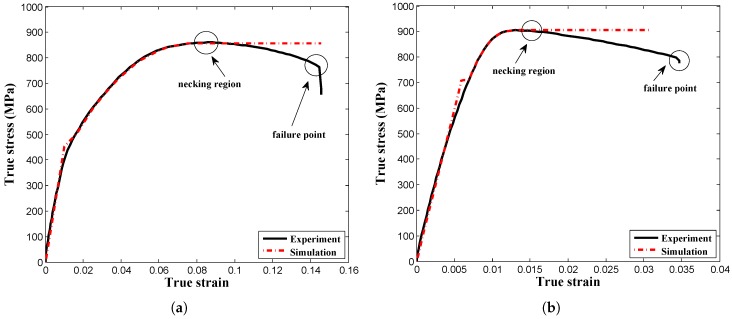

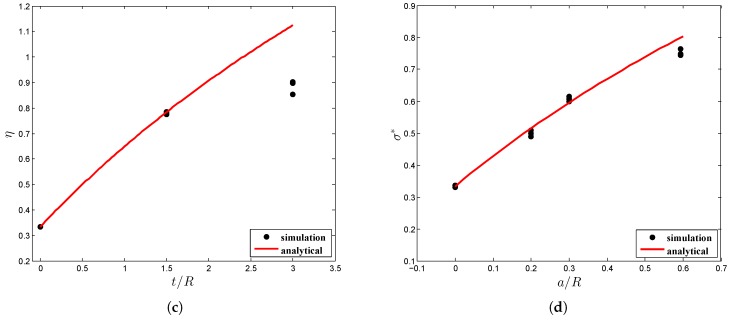
Flow stress curve at room temperature for AISI-1045 steel. (**a**) Comparison plot of the stress∼strain curve between experimental results and simulation results of the round bar specimen; (**b**) Comparison plot of the stress∼strain curve between experimental results and simulation results of the round bar notched specimen; (**c**) Comparison results of Bao and co-workers’ equation and numerical simulation; (**d**) Comparison results of the Bridgman equation and numerical simulation.

At first, the un-notched and pre-notched specimens were fabricated using the machine cutting process, and more than one sample was used to obtain the flow curve to get a better outcome in terms of the mechanical properties of the material in order to control the experimental error. A series of experiments including un-notched and pre-notched round bar, flat specimens were carried out simultaneously, as shown in Figure 7, at room temperature considering a wide range of quasi-static strain rate conditions to investigate the effect of stress triaxiality on the damage behavior of AISI-1045 medium carbon steel material. The flow curves accomplished from the tests were decomposed into elastic and plastic regions until necking for the identification of material properties as well as for the numerical model inputs. In addition, the mechanical properties of the material were determined carefully from performed experiments, because these properties needed to replicate the material’s behavior in the real situations as accurately as possible. blue Furthermore, the estimated properties were incorporated into commercial tools, and the work hardening behavior was simulated by considering the multi-linear isotropic hardening model for the assessment of stress triaxiality. The FE models were modeled using half symmetry conditions and meshed by different mesh sizes to reduce the computational time without affecting the accuracy, as shown in Figure 8a. From the outcome, a good correlation between experimental observations and the FE model was achieved in the elastic and plastic range until necking, as depicted in Figure 9. From Figure 9, it is evident that the estimation of mechanical properties from the experiments was done perfectly and can be further used to perform the estimation of stress triaxiality without any barrier. Subsequently, the stress components, such as, $\sigma_{1}$, $\sigma_{2}$, and $\sigma_{3}$, were chosen from the center of the cross-section of the specimen. Ductile damage mostly tends to occur at this region due to maximum stresses, as displayed in Figure 8b. Thus, the stress components were substituted into Equation (14) to determine the mean stress, $\sigma_{m}$, and the equivalent stress, $\sigma_{eq}$, and the set of stress triaxialities estimated for an entire specimens is listed in Table 3.

(13) $$\sigma^{*}=\frac{\left(\sigma_{1}+\sigma_{2}+\sigma_{3}\right)}{3\times \sqrt{0.5\times [\left(\sigma_{1}^{2}-\sigma_{2}^{2}\right)+\left(\sigma_{2}^{2}-\sigma_{3}^{2}\right)+\left(\sigma_{3}^{2}-\sigma_{1}^{2}\right)]}}$$

To verify the FE results of the flat specimens, Bao and co-workers’ formula was used as given below [26]:

(14) $$\eta =\frac{1}{3}+\sqrt{2}\;\ln\left(1+\frac{t}{4R}\right).$$

As depicted in Figure 9c, various stress triaxilities were calculated with respect to different thickness to radius ratios. From the comparison results, the estimated stress triaxialities from the numerical simulations were found to be considerably consistent with the analytical solutions. Also, stress triaxiality states according to Bridgman’s analytical model for the round bar specimens were employed to cross check the numerical data as follows [27]:

(15) $$\sigma^{*}=\frac{1}{3}+\ln\left(1+\frac{a}{2R}\right)$$

where $\sigma^{*}$, *R* and *a* are the stress triaxiality, the radius of the circumferential notch and the minimum cross section of the radius, respectively. From the data plotted in Figure 9d, it is evident that the numerical results aligned well with the analytical data. This aspect shows that the un-notched and pre-notched, and the round bar and flat shape specimen results are adequate for the estimation of JC damage model parameters.

Rearranging Equation (11) by neglecting the effects of strain rate and temperature, the failure model equation can be rewritten only in terms of the stress triaxiality effect with respect to fracture strain as follows [19]:

(16) $$\varepsilon_{f}=D_{1}+D_{2}\;\exp\left(D_{3}\sigma^{*}\right).$$

**Figure 10:**
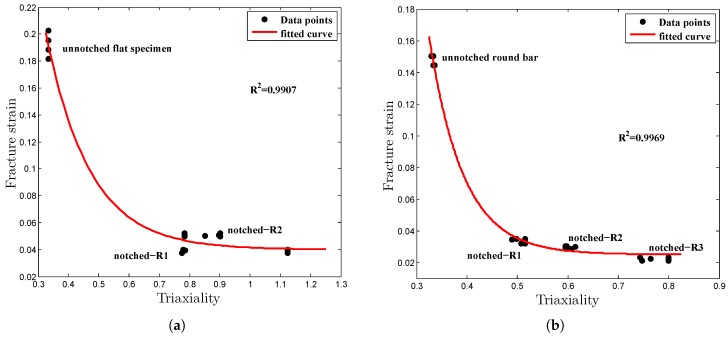
Relationship plot of strain to fracture and stress triaxiality. (**a**) Flat specimens; (**b**) Round bar specimens.

By substituting the stress triaxialities and corresponding fracture strain values into Equation (17), the relationship plot, $\varepsilon_{f}∼\sigma^{*}$, was developed, and from the coefficients of the fitted equation, as shown in Figure 10, the model parameters $D_{1}$, $D_{2}$ and $D_{3}$ were computed. Subsequently, the strain rate and the temperature dependent damage parameters $D_{4}$ and $D_{5}$ were obtained from two sets of high temperature and strain rate data by interpreting the failure strain variation [19]. The estimated JC fracture model parameters are outlined in Table 4, and the model parameters can be used in the metal forming applications to predict the ductile fracture behavior.

## 4. Discussion

**Figure 11:**
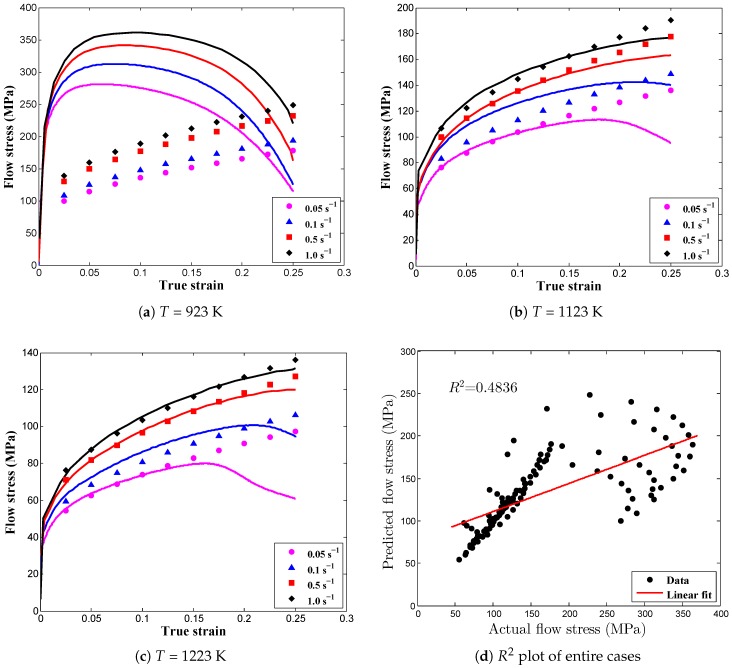
Comparison between experimental and predicted flow stress data using the modified Johnson-Cook model.

Numerous isothermal experiments were conducted over a practical range of deformation temperatures (650–950 ${}^{\circ }$C) and strain rates (0.05–1.0 s${}^{-1}$) to develop the JC material model to predict the flow stress data of AISI-1045 medium carbon steel. In addition, the experimental data obtained from the quasi-static strain rate tensile tests at room temperature were employed for the evaluation of damage model parameters. To verify the model adequacies and predictability, the proposed constitutive model predictions were compared with the experimental observations and were also incorporated into the numerical simulations for inverse calibrations. Figure 11 depicts the comparison of experimental stress-strain flow curves with the predicted flow curves by using the proposed JC model, whereas the model parameters were estimated from the optimization method. The data plotted in Figure 11 and the numerical data outlined in Table 5 clearly display that the presented optimized JC model is in good agreement with the experimental observations at higher temperatures for all strain rates, and on the contrary, the model cannot offer a better prediction of flow stress at the deformation temperature, 650 ${}^{\circ }$C, for all tested strain rates. Thus, from the prediction error comparison, the flow stress data obtained in the optimized JC model were found to be more consistent with the experimental data than the conventional JC model.

To perform the model evaluation, standard statistical measurements such as coefficient of determination ($R^{2}$) and an average absolute relative error (AARE) were adopted to quantify the proposed JC model predictability at discrete strains with an interval of 0.025 for all strain rates and temperatures. The $R^{2}$ provides information about the prediction strength of the linear relationship between the experimental observations and the predicted values, whereas AARE was estimated through a term-by-term comparison of the relative error. To perform this quantification, the following expressions were employed [28,29]:

(17) $$R^{2}=1-\frac{\sum_{i=1}^{n}{\left(\sigma_{\exp}^{i}-\sigma_{\mathrm{pred}}^{i}\right)}^{2}}{\sum_{i=1}^{n}{\left(\sigma_{\exp}^{i}-{\bar{\sigma }}_{\exp}\right)}^{2}},$$

(18) $$\mathrm{AARE}=\frac{1}{n}\sum_{i=1}^{n}\left|\frac{\sigma_{\exp}^{i}-\sigma_{\mathrm{pred}}^{i}}{\sigma_{\exp}^{i}}\right|\times 100\%,$$

where $\sigma_{\exp}$, $\sigma_{\mathrm{pred}}$, ${\bar{\sigma }}_{\exp}$ are the experimental flow stress, the predicted flow stress, and the mean flow stress, respectively, and *n* is the total number of data points.

**Figure 12:**
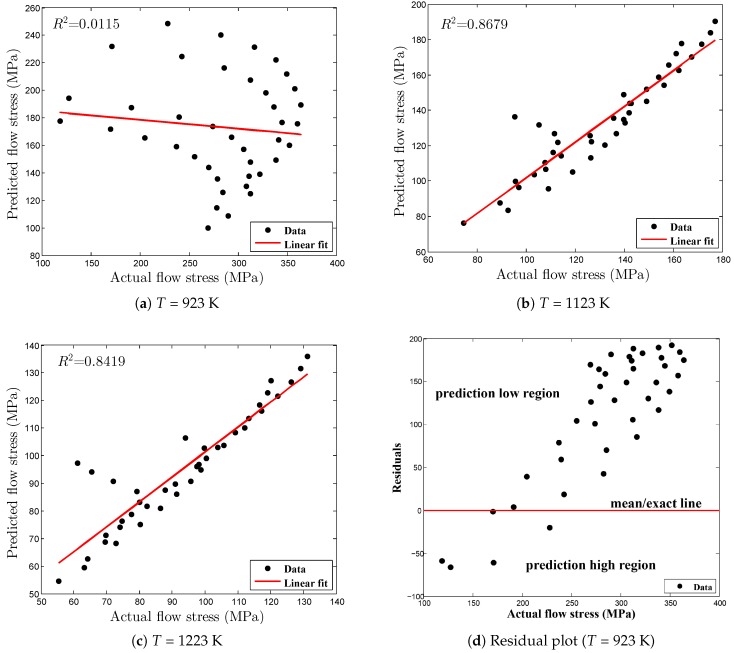
Relationship plots.

In this research, each test condition was examined by estimating the values of $R^{2}$ and AARE for each case rather than the traditional method, in which the entire data set was used to compute the statistical parameters as mentioned in Table 5. In this way, the prediction strength of the proposed JC model can be discussed in detail in terms of each and every test condition. The predicted flow curves and the graphical validation of the optimized JC model are shown in Figure 11 for all strain rates and deformation temperatures. Figure 11a depicts the comparison plot between predicted flow curves and experimental data and it shows that the developed model overpredicts the flow stress data. The numerical values $R^{2}$ and AARE were found to be 0.0012, lacking the prediction of the linear relationship as illustrated in Figure 12a, and 40.9341, respectively. From these numbers, it is clear that the optimized JC model cannot predict the material behavior at a deformation temperature of 923 K for all strain rate conditions. However, somehow, as shown in Figure 12a, there is some abnormal behavior in the distribution of data points. To verify this phenomenon, the residual plot, decomposed into three parts—low, exact, and high predictions—is plotted in Figure 12d. From Figure 12d, it is clearly shown that the proposed model mostly under predicts the flow stress, as the most of the data points were distributed linearly, somehow in exponential form, above the exact prediction line. This phenomenon explains that by adding some noise function into a negative linear or exponential form to the original flow stress model, this prediction error can be avoided. Likewise, Figure 11b shows that at a deformation temperature of 1123 K for all strain rates, it is evident that most of the predicted flow stress data are close to the experimental observations, whereas Figure 12b exhibits a good correlation between actual and predicted data. In addition, the computed corresponding values of the statistical parameters, $R^{2}$, 0.8679, and AARE, 5.9313%, show that the proposed JC model has considerable potential to predict the flow stress under the tested conditions. Furthermore, Figure 11c and Figure 12c illustrate the good correlation between experimental and predicted data under the tested conditions. $R^{2}$ and AARE were found to be 0.8419 and 5.9689%, respectively. Besides, it is noted that the prediction error minimization considering the material parameters, *c* and *m* using the optimization procedure led to significant improvement in the JC model prediction. For all test conditions, the overall AARE reduced from 18.12% to 17.61%. The differences between the models may be small but this small error can cause the false estimation of flow stress which leads to the inaccurate prediction of material behavior.

Overall, it was observed that the optimized JC model for AISI-1045 medium carbon steel can be used for flow stress prediction at high temperatures over the entire tested range of strain rates. Even though the overall flow stress prediction was good, in a few cases, for example, at deformation temperature 1123 K and at a strain rate of 0.05 s${}^{-1}$, deviation was found to occur. The reason for the deviation is mainly because of the softening behavior and the drop in flow stress that happens in the early stages at the following deformation temperatures: 923 K (at 0.05 s${}^{-1}$∼1.0 s${}^{-1}$), 1123 K (at 0.05 s${}^{-1}$) and 1223 K (at 0.05 s${}^{-1}$). The decreased flow stress values led to the improper estimation of the model parameters, because the JC model is just a phenomenological model that does not consider any of the material physical aspects. In addition, sometimes numerical numbers such as $R^{2}$ can lead to error, even though the model is adequate numerically. So, in order to remove this misinterpretation, graphical validation is necessary, and if both numerical and graphical outputs are admissible, then the developed flow stress model is good to use for future the calculations.

## 5. Conclusions

In this paper, numerous isothermal hot tensile tests at elevated temperatures (650–950 ${}^{\circ }$C) and strain rates (0.05–1.0 s${}^{-1}$) were carried out to identify the Johnson-Cook material model parameters for AISI 1045 medium carbon steel. The nonlinear programming solver fmincon-based optimization procedure was employed for minimizing the prediction error between the experiments and the predictions to improve the ability of the proposed JC constitutive model. Overall, the results obtained from the optimized JC model showed better agreement with the experimental observations than those of the traditional method. However, more computational time is required to achieve the JC material constants while performing the optimization procedures than with the conventional method. Besides, the developed model predictability was evaluated in terms of the metrics $R^{2}$ and AARE. In addition, using the commercial tool, the numerical simulations were modeled extensively in order to develop the damage model using the experimental observations obtained from the quasi-static tensile tests with smooth and notched specimens at room temperature. The numerical results displayed a good agreement with the corresponding experimental data. From the discussion, it was found that the JC material model requires less effort to predict the model parameters and, on the contrary, the JC damage model requires numerous experimental data to find the model parameters, which necessitates extensive time and cost efforts. Besides, the prediction error between the experimental and predicted data ensures that the proposed constitutive model is credible at elevated temperatures and higher strain rates. Based on these research outcomes, the detailed identification of the JC material and damage model can be devised using the procedure presented here to predict material ductile fracture behavior.

## Figures and Tables

**Table 1:** Chemical composition of AISI-1045 medium carbon steel (in wt.%).

C	Fe	Mn	P	S
0.42–0.50	98.51–98.98	0.60–0.90	≤0.04	≤0.05

**Table 2:** Johnson-Cook material model parameters of AISI-1045 steel.

Regular JC Model					Optimized JC Model				
*A* (MPa)	*B* (MPa)	*n*	*C*	*m*	*A* (MPa)	*B* (MPa)	*n*	*C*	*m*
50.103	176.091	0.5176	0.1056	0.5655	50.103	176.091	0.5176	0.095	0.6622

**Table 3:** Different stress triaxiality data determined from numerical simulations using experimental data from round bar, flat un-notched and pre-notched specimens.

T	$\dot{\varepsilon }$ (s${}^{-1}$)	Flat 6w	N-R0	N-R1	N-R2	N-R3
27 ${}^{\circ }$C	0.001	0.3345	–	0.8523	0.7756	–
	0.005	0.3337	–	0.9017	0.7782	–
	0.010	0.3332	–	0.8961	0.7852	–
	0.050	0.3333	–	0.9015	0.7829	–
	0.0001	–	0.3359	0.5082	0.6070	0.7473
	0.001	–	0.3299	0.4989	0.6152	0.7641
	0.010	–	–	0.4897	0.5983	0.7438

**Table 4:** Johnson-Cook fracture model parameters of AISI-1045 steel.

Round Bar and Notched Specimens					Flat and Notched Specimens				
*D1*	*D2*	*D3*	*D4*	*D5*	*D1*	*D2*	*D3*	*D4*	*D5*
0.025	16.93	-14.8	0.0214	0	0.04	1.519	-6.905	-0.023	1.302

**Table 5:** Different stress triaxiality data obtained from experiments.

Conditions	$R^{2}$	Overall-$R^{2}$	AARE (%)	Overall-AARE (%)
923 K	0.0115	0.4836	40.9341	17.6112
1123 K	0.8679		5.9313	
1223 K	0.8419		5.9689	
