# Correction: Murugesan, M., et al. Johnson Cook Material and Failure Model Parameters Estimation of AISI-1045 Medium Carbon Steel for Metal Forming Applications. *Materials* 2019, *12*, 609

**DOI:** 10.3390/ma13245744

**Published:** 2020-12-16

**Authors:** Mohanraj Murugesan, Dong Won Jung

**Affiliations:** Department of Mechanical Engineering, Jeju National University, Jeju-Do 63243, Korea; mohanaero45@gmail.com

The author wishes to make the following corrections to this paper [1].

1. Due to the first author’s misunderstanding about the meaning of the equal contribution during the submission time, remove the “†” symbol from both authors’ names with it’s explanation “These authors contributed equally to this work”.
2. Due to the wrong sub-caption in Figure 2d, replace
  (d) *T* = 650 °C
  with
  (d) *T* = 950 °C
3. Due to the wrong caption in Table 5, replace
  Table 5. Different stress triaxiality data obtained from experiments.
  with
  Table 5. Statistical measurements of the optimized JC model.

These changes have no impact on the discussion and conclusions of the research paper. The authors would like to ask for a sincere apology for any inconvenience caused to the readers by these changes.
